# Supplementary material for: Integrating pheromonal and spatial information in the amygdalo-hippocampal network
Source: Nat Commun. 2021 Sep 6;12:5286. doi: 10.1038/s41467-021-25442-5 (PMC8421364; doi:10.1038/s41467-021-25442-5)
Supplement: Supplementary file 1 — Supplementary Information [file 41467_2021_25442_MOESM1_ESM.pdf]

Supplementary Figure 1.

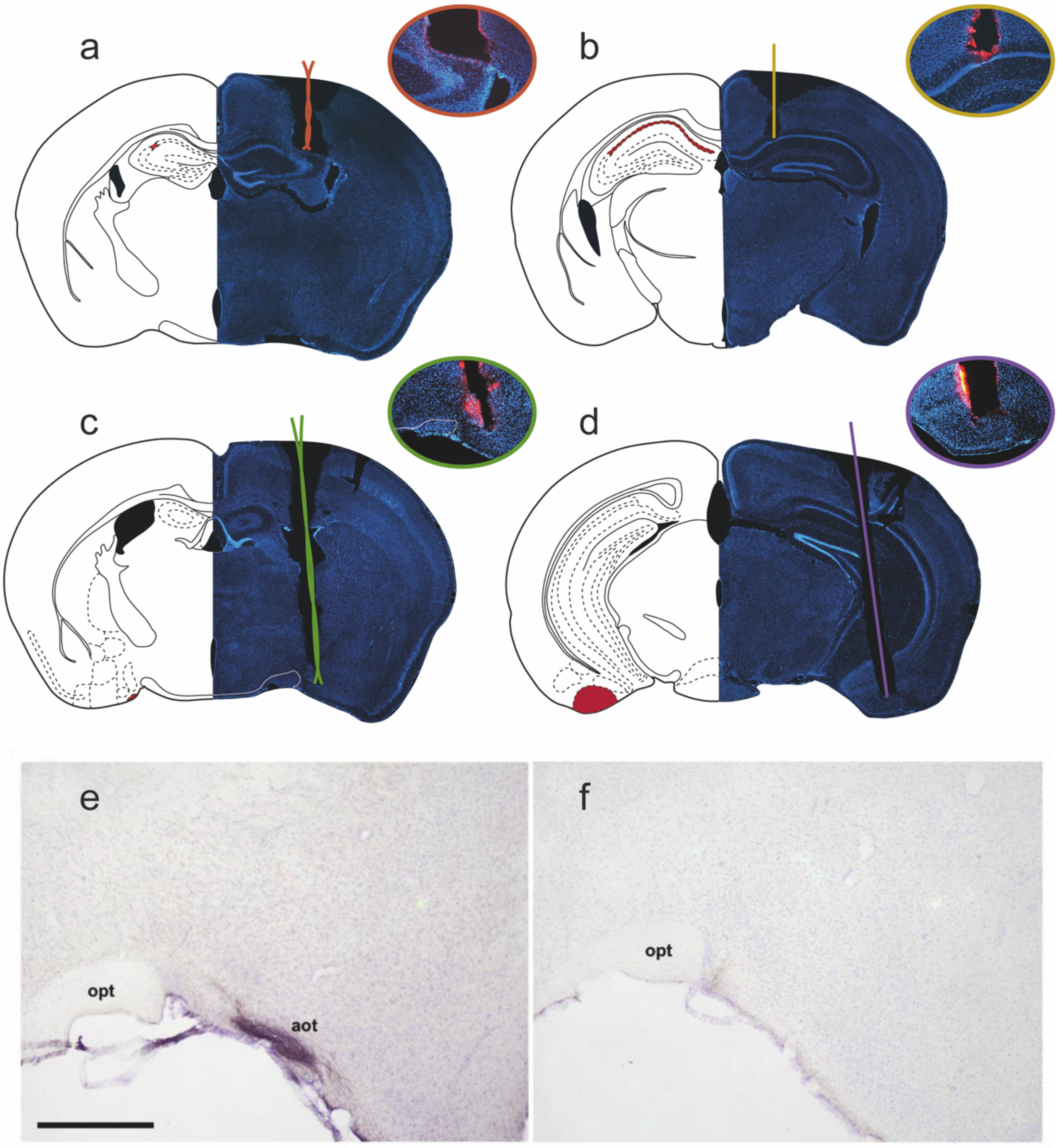

Supplementary Figure 1. Semischematic representation of electrodes location in LTP experiments. Bipolar stimulation electrodes were positioned in the dorsal hippocampal Schaffer collaterals (a) and in the accessory olfactory tract (aot). (c), whereas monopolar register electrodes were positioned in the dorsal hippocampal CA1 pyramidal cell layer (b) and in the posteromedial cortical amygdaloid nucleus (d). Targeted structures are highlighted in red in the schemes. Photomicrographs of DAPI-stained sections show placement of the electrodes and higher magnification inset is provided for each case showing DiI-stained electrode path. Transverse sections through the telencephalon of mice injected with anterograde neural tracers in the accessory (e) and in the main olfactory bulb (f), showing the pathway of the fibers of the aot at the level of the anterior amygdaloid area, compared to the very sparse olfactory projection present at this same level. AOB injections, n = 6; MOB injections, n = 6, Scale bar in (e), valid for f, 500 μm.

Supplementary Figure 2.

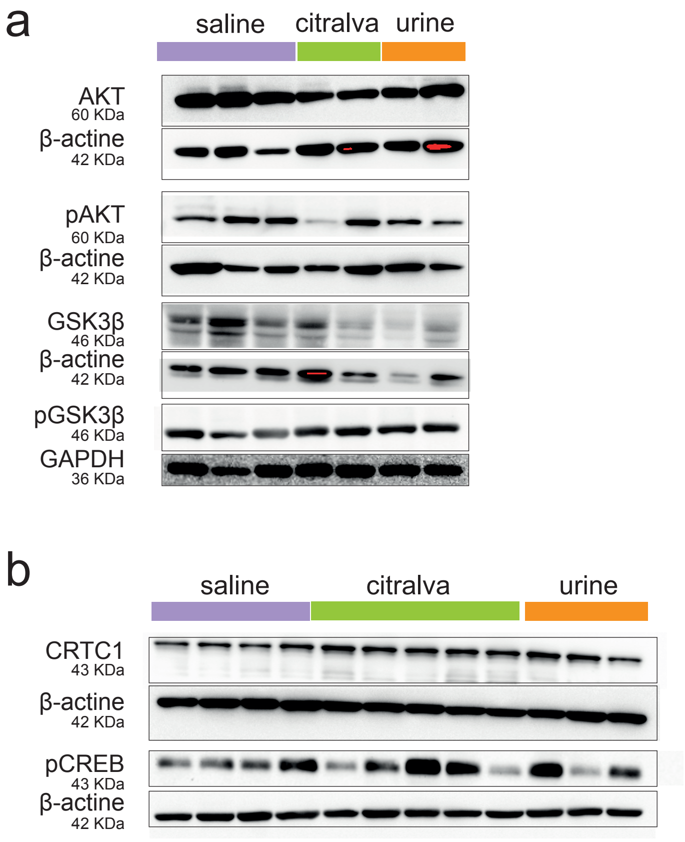

Supplementary Figure 2. Complement of the Figure 6d. (a) Western-blots of AKT and GSK3β for CA1 samples. Six additional cases (shown in boxplots in Figure 6c) were run in a second gel; the first lane of each gel corresponds to first lane in Figure 6d. (b) Western-blots of CRTC1 and pCREB for PMCo samples. No significant differences between groups were found. For both the CA1 and PMCo experiments, n = 6 animals per group were used (total n = 18)

Supplementary Figure 3.

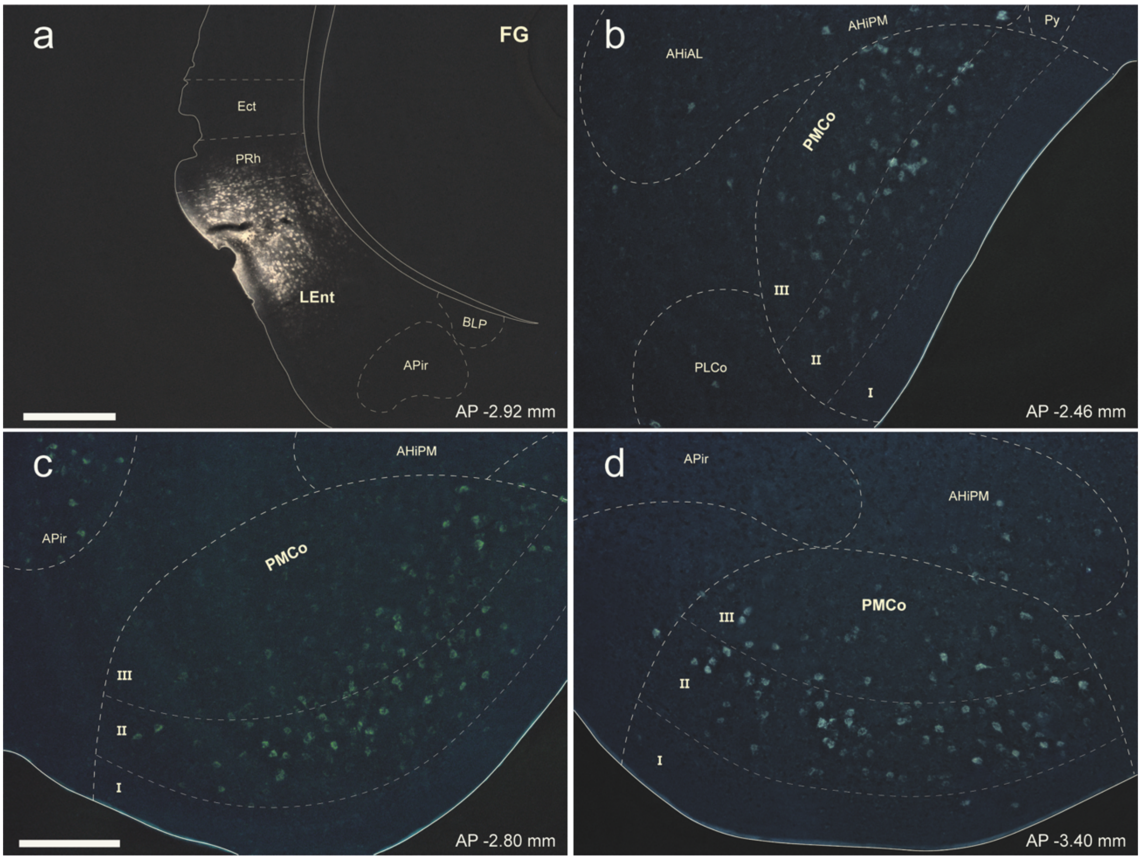

Supplementary Figure 3. **FluoroGold retrograde tracing confirms direct neuroanatomical connection between the dlEnt and the PMCo**. (a). FluoroGold restricted injection into the dlEnt (n = 4), at an anteroposterior level where anterograde labeled fibers were present after TBDA injection in the PMCo. (b), (c), (d). Photomicrographs showing retrograde labeled somata through different PMCo rostrocaudal coordinates, confirming direct output from all the PMCo extent to the dlEnt. Scale Bar a, 500 μm; c, valid for b, d, 100 μm.

Supplementary Table 1

LTP induction by tetanic and urine stimulation

area	stimulus	time	*p*-value
PMCo	tetanic	5-10	0.1195
		10-20	0.0015
		20-25	0.0027
	urine	5-10	0.2959
		10-20	0.0150
		20-25	<0.0001
CA1	tetanic	5-10	0.0389
		10-20	0.1153
		20-25	0.0001
	urine	5-10	0.0047
		10-20	0.0002
		20-25	0.0008

Multiple comparisons after Friedman test, against the basal value (Dunn's test) in the three time periods analyzed from the measures of LTP corresponding with Figure 4.

LTP induction in zinc sulfate lesioned animals

area	stimulus	time	*p*-value
PMCo	citralva	5-10	>0.9999
		10-20	>0.9999
		20-25	>0.9999
	urine	5-10	0.0550
		10-20	0.0244
		20-25	0.0014
CA1	citralva	5-10	>0.9999
		10-20	>0.9999
		20-25	>0.9999
	urine	5-10	0.0078
		10-20	0.0078
		20-25	0.0078

Multiple comparisons after Friedman test, against the basal value (Dunn's test) in the three time periods analyzed from the measures of LTP corresponding with Figure 5.

Supplementary Table 2

Statistical correlations between the time spent investigating male-soiled bedding and the c-fos expression in PMCo, dLEnt and CA1

		Behav.	PMCo	Ent	CA1
**Behav.**	Pearson correlation	1	0.839*	0.862*	0.253
	Sig. (bilateral)		0.037	0.027	0.628
	N		6	6	6
**PMCo**	Pearson correlation		1	0.903*	0.288
	Sig. (bilateral)			0.014	0.579
	N			6	6
**Ent**	Pearson correlation			1	0.166
	Sig. (bilateral)				0.753
	N				6

The time spent by females exploring male bedding showed a significant statistical correlation with c-fos levels in PMCo (data from [29]) and dLEnt. No significant correlations were observed between the CA1 c-fos expression and the other variables. Although the lack of correlation with c-fos expression in CA1 is surprising, it could be explained by the fact that the number of c-fos expressing cells in the dorsal CA1 is very low (only a few scattered pyramidal neurons were labeled), thus making it difficult to reveal a statistical correlation. In any case, the strong correlation between the time spent exploring male pheromones and the Fos levels in PMCo and dorsal dLEnt cortex suggests that indeed vomeronasal information is relayed from the PMCo to the entorhinal cortex. * $p < 0.05$, bilateral.
