# Supplementary material for: Integrating pheromonal and spatial information in the amygdalo-hippocampal network
Source: Nat Commun. 2021 Sep 6;12:5286. doi: 10.1038/s41467-021-25442-5 (PMC8421364; doi:10.1038/s41467-021-25442-5)
Supplement: Supplementary file 3 — Description of Additional Supplementary Files [file 41467_2021_25442_MOESM3_ESM.pdf]

Description of Additional Supplementary Files

File Name: Supplementary Movie 1

Description: **Virtual reality system set up.** Navigation through corridor 3 of the virtual environment. Visual cues are present in sector 2, separated from sector 3 by a black curtain that automatically opens when the animal gets close. In sector 3, a cotton swab is automatically directed to the animal's nose until it is close enough to allow contact. The cotton swab withdraws at the end of sector 3. A **+** sign indicates the reward obtained by the animal at the end of sector 4. The video is recorded with forced movement of the wheel without the presence of an animal. A higher magnification detail of the system is shown in the right panel, which displays the approach of the cotton swab towards the animal's nose.
