# Supplementary material for: Integrating pheromonal and spatial information in the amygdalo-hippocampal network
Source: Nat Commun. 2021 Sep 6;12:5286. doi: 10.1038/s41467-021-25442-5 (PMC8421364; doi:10.1038/s41467-021-25442-5)
Supplement: Supplementary file 5 — Reporting Summary [file 41467_2021_25442_MOESM5_ESM.pdf]

nature portfolio

Corresponding author(s): Enrique Lanuza / Vicent Teruel-Martí

Last updated by author(s): Aug 2, 2021

Reporting Summary

Nature Portfolio wishes to improve the reproducibility of the work that we publish. This form provides structure for consistency and transparency in reporting. For further information on Nature Portfolio policies, see our Editorial Policies and the Editorial Policy Checklist.

Statistics

For all statistical analyses, confirm that the following items are present in the figure legend, table legend, main text, or Methods section.

n/a	Confirmed	
☐	☒	The exact sample size (n) for each experimental group/condition, given as a discrete number and unit of measurement
☐	☒	A statement on whether measurements were taken from distinct samples or whether the same sample was measured repeatedly
☐	☒	The statistical test(s) used AND whether they are one- or two-sided<br>*Only common tests should be described solely by name; describe more complex techniques in the Methods section.*
☒	☐	A description of all covariates tested
☐	☒	A description of any assumptions or corrections, such as tests of normality and adjustment for multiple comparisons
☐	☒	A full description of the statistical parameters including central tendency (e.g. means) or other basic estimates (e.g. regression coefficient) AND variation (e.g. standard deviation) or associated estimates of uncertainty (e.g. confidence intervals)
☐	☒	For null hypothesis testing, the test statistic (e.g. F, t, r) with confidence intervals, effect sizes, degrees of freedom and P value noted<br>*Give P values as exact values whenever suitable.*
☒	☐	For Bayesian analysis, information on the choice of priors and Markov chain Monte Carlo settings
☒	☐	For hierarchical and complex designs, identification of the appropriate level for tests and full reporting of outcomes
☐	☒	Estimates of effect sizes (e.g. Cohen's d, Pearson's r), indicating how they were calculated

Our web collection on statistics for biologists contains articles on many of the points above.

Software and code

Policy information about availability of computer code

Data collection	Open Ephys GUI v0.5.4 (http://open-ephys.org). Signal v6.0 (Cambridge Electronics Design). DeepLabCut v2.1.10.2 (http://www.mackenziemathislab.org/deeplabcut). Unity v2018.4.36 (https://unity3d.com/get-unity/download/archive).
Data analysis	MATLAB v20.0 (MathWorks). R studio (1.4.1106). Python v3.0 (https://www.python.org). Scikit-learn python package v0.17 (https://scikit-learn.org/0.17/). MVGC Multivariate Granger Causality Matlab® Toolbox (https://github.com/SacklerCentre/MVGC1). FIJI v2.1.0 (https://imagej.net/software/fiji/). ImageGauge v4.0 (Fuji Film Life Science).

For manuscripts utilizing custom algorithms or software that are central to the research but not yet described in published literature, software must be made available to editors and reviewers. We strongly encourage code deposition in a community repository (e.g. GitHub). See the Nature Portfolio guidelines for submitting code & software for further information.

Data

Policy information about availability of data

All manuscripts must include a data availability statement. This statement should provide the following information, where applicable:

- Accession codes, unique identifiers, or web links for publicly available datasets
- A description of any restrictions on data availability
- For clinical datasets or third party data, please ensure that the statement adheres to our policy

The raw data of the LFP recordings of the experiments in the virtual reality setup generated in this study are available in the Zenodo database under accession code https://doi.org/10.5281/zenodo.5153116.
The data generated in the LFP experiments (causality measures and theta components), long term potentiation experiments, behavioural results, protein quantification using western blots, and c-Fos quantification are provided in the Source Data file.

Field-specific reporting

Please select the one below that is the best fit for your research. If you are not sure, read the appropriate sections before making your selection.

- [x] Life sciences
- [ ] Behavioural & social sciences
- [ ] Ecological, evolutionary & environmental sciences

For a reference copy of the document with all sections, see nature.com/documents/nr-reporting-summary-flat.pdf

Life sciences study design

All studies must disclose on these points even when the disclosure is negative.

Sample size	No statistical method was explicitly used to determine the sample size prior to the study. The number of experiments was consistent with the research works from which we were inspired (LTP experiments, Strauch & Manahan-Vaughan, 2020; c-fos measures, Moncho-Bogani et al., 2005). The selected sample size was justified and approved by the Research Ethics and Animal Welfare Committee of the University of Valencia, that granted authorization for the experiments.
Data exclusions	All criteria for data exclusion were decided upon prior to data collection. In the behavioral experiments, mice that showed an initial preference for one part of the arena were removed from the experiment. Experiments with off-target placement of electrodes were excluded from our analyses. For LTP experiments, recordings were discarded if there were artifacts or a low amplitude evoked potential. For the electrophysiological experiments, the records were excluded if any of the channels showed a distorting noise level.
Replication	The same experimental conditions were guaranteed in each of the procedures. We observed similar results which satisfied the same statistical criteria across experiments. Dispersion of data indicated acceptable replication; we have plotted independent data points as a manner to show the consistency of the data.<br>In those experiments in which urethane anesthesia was used (LTP), we monitored the presence of characteristic neural oscillations by local field potential recordings on dorsal hippocampus. LTP measurements were performed when the levels of theta rhythmicity were prominent after a tail-pinch stimulus, as a sign of hippocampal response under an acceptable level of anesthesia.
Randomization	Animals were randomly assigned numbers and tested blind for the experimental conditions.
Blinding	The experiments were based on the presence of olfactory stimuli. For this reason, it was difficult to ensure the condition of blind experimenter. However, the same stimuli led to similar results, and the difference between stimuli showed substantial differences. Moreover, the action of the experimenter was limited to exposing the swab with the olfactory stimulus; in LTP or animal tracking measurements, the automated methods of data collection avoided the experimenter's influence.

Reporting for specific materials, systems and methods

We require information from authors about some types of materials, experimental systems and methods used in many studies. Here, indicate whether each material, system or method listed is relevant to your study. If you are not sure if a list item applies to your research, read the appropriate section before selecting a response.

Materials & experimental systems

n/a	Involved in the study	
[ ]	[x]	Antibodies
[x]	[ ]	Eukaryotic cell lines
[x]	[ ]	Palaeontology and archaeology
[ ]	[x]	Animals and other organisms
[x]	[ ]	Human research participants
[x]	[ ]	Clinical data
[x]	[ ]	Dual use research of concern

Methods

n/a	Involved in the study	
[x]	[ ]	ChIP-seq
[x]	[ ]	Flow cytometry
[x]	[ ]	MRI-based neuroimaging

Antibodies

Antibodies used	The following primary antibodies were used: 1) pAkt (9271, Cell Signaling Technology), 2) Akt (9272, Cell Signaling Technology), 3) CRTC1 (2587, Cell Signaling Technology), 4) pCreb (9191, Cell Signaling Technology), 5) GSK3β (JM-3494-100, MBL), 6) pGSK3β-Ser9 (05-643, Merk), 7) β-Actin (A1978, Sigma Aldrich), 8) vGLUT1 (135302, Synaptic Systems), 9) Fluorogold (AB153-I, Millipore), 10) reelin (MAB5364, Millipore), 11) calbindin (CB38, SWANT), 12) c-Fos (SC-52, Santa Cruz Biotech).<br>Secondary antibodies were: anti-mouse IgG H&L Chain Specific Peroxidase Conjugate (401215, Calbiochem) or anti-rabbit IgG HRP-linked (7074S, Cell Signaling), Alexa 488-conjugated goat anti-rabbit (111-545-003, Jackson Inmunoresearch), Rhodamine RedTM-X goat-anti mouse IgG (R6393, Life Technologies).
Validation	All antibodies used in this study are commercially available and validated by the manufacturers.<br>1) Validation of anti-phospho-Akt (Ser473): this antibody does not recognize Akt with an alanine substituion at Ser473 (https://www.cellsignal.com/datasheet.jsp?productId=9271&images=1)

2) Validation of anti-Akt: western-blot labeling with this antibody was abolished in CHO cells in which Akt expression was silenced using an Akt siRNA (https://www.cellsignal.com/products/primary-antibodies/akt-antibody/9272)
3) Validation of anti-CRTC1: The specificity of this antibody was verified using CRTC1 knockdown with CRISPR interference in neurons (Esvald et al, J Neurosci. 2020;40:1405-26. doi:10.1523/JNEUROSCI.0367-19.2019)
4) Validation of anti-pCreb: it detects endogenous levels of CREB only when phosphorylated at serine 133. This antibody also detects the phosphorylated form of the CREB-related protein ATF-1. Extensively validated in the literature: https://www.cellsignal.com/products/primary-antibodies/phospho-creb-ser133-antibody/9191
5) Validation of anti-GSK3β: it detects a single band of the expected molecular weight in western-blot (https://www.mblintl.com/products/jm-3494-100/)
6) Validation of anti-pGSK3β-Ser9: it detects a single band of the expected molecular weight in western-blot https://www.merckmillipore.com/ES/es/product/Anti-phospho-GSK3-Ser9-Antibody-clone-2D3,MM_NF-05-643
7) Validation of anti-β-Actin: Mouse Monoclonal antibody that recognizes an epitope located on the N-terminal end of the β-isoform of actin. It detects a single band of the expected molecular weight in western-blot (https://www.sigmaaldrich.com/ES/es/product/sigma/a1978)
8) Validation of anti-vGLUT1: validated in brain lysates of a vGlut1 KO strain (https://sysy.com/product/135302#gallery-2)
9) Validation of anti-Fluorogold: highly specific antibody that gives no signal if tissue has not been previously treated/injected with Fluorogold. (https://www.merckmillipore.com/ES/es/product/Anti-Fluorescent-Gold-Antibody,MM_NF-AB153-I)
10) Validation of anti-reelin; monoclonal antibody that detects a single band of the expected molecular weight in western-blot (https://www.merckmillipore.com/ES/es/product/Anti-Reelin-Antibody-a.a.-164-496-mreelin-clone-G10,MM_NF-MAB5364). It gives no staining in the reeler mouse (a mouse with a large mutation within the reelin protein) (Armstrong et al. J Comp Neurol. 2011;519(8):1476-1491. doi:10.1002/cne.22577)
11) Validation of anti-calbindin: extensively validated antibody in the literature (see http://antibodyregistry.org/AB_10000340). It recognizes a 28-kDa band in Western blots
12) Validation of anti-cFos: extensively validated antibody in the literature (see http://antibodyregistry.org/AB_2106783). Characterized by preadsorption tests in western blot and immunohystochemistry (Gaszner et al. 2007. J Comp Neurol. 500(4):708-19. doi: 10.1002/cne.21177. PMID: 17154253)

Animals and other organisms

Policy information about studies involving animals; ARRIVE guidelines recommended for reporting animal research

Laboratory animals	Subjects were adult CD1 male (n=20) and female (n=76) mice (2-5 months of age, 30 – 60 g; animal facility of the Central Unit of Research, University of Valencia, Valencia, Spain). Mice were housed in groups of 2-4 mice per cage on a 12 h light/dark cycle with constant ambient temperature (22 ± 1°C) and humidity, and water and food available ad libitum.
Wild animals	The study did not involve wild animals.
Field-collected samples	The study did not involve samples collected from the field
Ethics oversight	All experimental procedures were approved by the Research Ethics and Animal Welfare Committee of the University of Valencia, in accordance with the European Communities Council Directive (2010/63/UE) on the protection of animals used for scientific purposes.

Note that full information on the approval of the study protocol must also be provided in the manuscript.
